# Salt Use Behaviours of Ghanaians and South Africans: A Comparative Study of Knowledge, Attitudes and Practices

**DOI:** 10.3390/nu9090939

**Published:** 2017-08-28

**Authors:** Elias Menyanu, Karen E. Charlton, Lisa J. Ware, Joanna Russell, Richard Biritwum, Paul Kowal

**Affiliations:** 1 School of Medicine, Faculty of Science, Medicine and Health, University of Wollongong, Northfields Avenue, Wollongong, NSW 2522, Australia; ekm@uowmail.ed.au; 2 Illawarra Health and Medical Research Institute, Wollongong, NSW 2522, Australia; 3 Hypertension in Africa Research Team (HART), North-West University, Potchefstroom 2531, South Africa; lisa.jayne.ware@gmail.com; 4 School of Health and Society, Faculty of Social Sciences, University of Wollongong, Northfields Avenue, Wollongong, NSW 2522, Australia; jrussell@uow.edu.au; 5 Department of Community Health, University of Ghana, Accra, Ghana; biritwum@africaonline.com.gh; 6 World Health Organization Study on global AGEing and adult health (SAGE), 1211 Geneva, Switzerland; kowalp@who.int; 7 Research Centre for Generational Health and Ageing, University of Newcastle, Newcastle, NSW 2300, Australia; 8 Department of Anthropology, University of Oregon, Eugene, OR 97403, USA

**Keywords:** discretionary salt, dietary salt, sodium, health behaviour, blood pressure

## Abstract

Salt consumption is high in Africa and the continent also shares the greatest burden of hypertension. This study examines salt-related knowledge, attitude and self-reported behaviours (KAB) amongst adults from two African countries—Ghana and South Africa—which have distributed different public health messages related to salt. KAB was assessed in the multinational longitudinal World Health Organisation (WHO) study on global AGEing and adult health (WHO-SAGE) Wave 2 (2014–2015). Respondents were randomly selected across both countries—Ghana (*n* = 6746; mean age 58 years old; SD 17; 41% men; 31% hypertensive) and South Africa (*n* = 3776, mean age 54 years old; SD 17; 32% men; 45% hypertensive). South Africans were more likely than Ghanaians to add salt to food at the table (OR 4.80, CI 4.071–5.611, *p* < 0.001) but less likely to add salt to food during cooking (OR 0.16, CI 0.130–0.197, *p* < 0.001). South Africans were also less likely to take action to control their salt intake (OR 0.436, CI 0.379–0.488, *p* < 0.001). Considering the various salt reduction initiatives of South Africa that have been largely absent in Ghana, this study supports additional efforts to raise consumer awareness on discretionary salt use and behaviour change in both countries.

## 1. Introduction

The Global Burden of Disease study has demonstrated that diet contributes significantly to risk of non-communicable diseases (NCDs) such as cancer, cardiovascular disease (CVD) and diabetes [1]. Dietary risk factors include diets low in fruits, vegetables, whole grains, nuts and seeds, fibre, omega-3 oils, and polyunsaturated fatty acids and diets that are high in sodium, red meat, processed meat, sweetened beverages, and trans fats [2,3,4,5,6]. The nutrition transition in many Sub-Saharan countries is resulting in a change in dietary patterns from traditional, plant-based diets to increasing intakes of processed foods that tend to be high in sugar and/or fat, known as energy dense, nutrient poor (EDNP) foods that are generally also high in salt [7]. Excess salt intake has been identified as one of the leading global health risks [8] and population level salt reduction is recognized as a cost-effective means of reducing blood pressure [9,10,11,12] and, in turn, reducing the risk of heart disease and stroke [13,14]. A 30% reduction in population level salt by 2020 is one of the voluntary global health targets identified by the World Health Organization [7]. 

On a global scale, it has been estimated that 1.7 million lives could be saved annually if salt consumption levels were decreased to recommended levels of less than 5g per day [15]. Across high and low-middle income countries, interventions to reduce population salt intake are considered cost effective (less than $1USD per person per year) [16,17].

Over 75% of cardiovascular deaths take place in low and middle-income countries [18] with the African region estimated to have around 20 million people with CVD [18]. As such, Africa shares the greatest burden of hypertension with almost half of adults aged 25 and older diagnosed and potentially more adults with undiagnosed, untreated and uncontrolled hypertension [19]. In Ghana, the prevalence of hypertension has continued to rise over the past 40 years [20,21,22]. South Africa (SA) equally shares a large burden of hypertension [23,24]. The direct healthcare costs attributable to non-optimal blood pressure in Sub-Saharan Africa (SSA) in 2001 were estimated to be two billion US dollars [25].

More than two-thirds of African populations attach low importance to dietary salt reduction as a significant approach to addressing hypertension [26,27]. In most African countries, salt is commonly added to food at the table and during cooking, and is a major ingredient found in commonly used sauces and seasonings [28,29]. Salted fish and meat are eaten frequently [30] while bread contains levels of salt that are generally higher than in countries in Europe and North America [31,32]. In SA, other sources of salt include cereals, meat and meat products, milk and dairy products, processed meats, meat pies and margarine. In that country, it is estimated that discretionary salt intake accounted for almost half of the total dietary intake in a sample of black urban dwelling people [33]. This represents a greater contribution as compared to many high income countries, where 75–85% of dietary salt intake is estimated to come from processed foods [34]. The Ghana Demographic and Health Survey indicated that 84% of women surveyed reported that someone in their household had consumed processed foods containing salt within the past 24 hours, while more than a third had consumed salted dried fish, 21% reported having had canned fish, meat and legumes and 24% reported the use of other processed foods containing salt [35]. The report identified a high use of salty foods in both rural and urban areas. Similarly, the South African Demographic and Health Survey 2003 indicated that more than 30% of survey respondents reported adding salt to food at the table and consuming salty snacks more than twice a week [36].

To reduce discretionary salt consumption in SA, there has been a concentrated focus on consumer education and awareness in recent years [37]. In addition, in June 2016, the SA government implemented mandatory legislation related to maximum levels of salt permitted in a wide range of processed food categories, including breads, meats, cereal products, fat spreads, snack foods and savoury products [38]. These foods have previously been shown to contribute significantly to overall non-discretionary salt intake in the South African population [33]. In contrast, in Ghana, there have been no concerted efforts by government or non-governmental organizations to implement salt reduction strategies. Instead, there has been a focus on the prevention of iodine deficiency through universal salt iodisation programmes [39,40,41] with the message to consume iodized salt widely disseminated through the mass media [39]. Public health concerns related to the association between increased salt intake and cardiovascular risk in Ghana have received comparatively little attention despite promising proof of concept studies [42]. Iodisation programmes and salt reduction strategies are not mutually exclusive, as has been demonstrated [43]. However, for both programmes to successfully coexist, different sectors of government (nutrition and NCDs) need to work together, to effectively monitor the iodine status of populations as salt content in the food supply decreases. 

Given that the population in Ghana has been urged to consume iodised salt [39,40,44] combined with the generally increased accessibility of EDNP processed foods, it is timely to investigate knowledge, attitudes and behaviours (KAB) related to salt use in Ghana and to compare these against SA, an African country in which salt reduction has been strongly emphasized. Ghana and SA share similar socio-demographic characteristics, as well as a high hypertension burden. The findings will be important—particularly for low and middle-income countries—for informing approaches to reducing the health risks through a better understanding of salt intake behaviours.

## 2. Materials and Methods

### 2.1. Study Design

Analysis for this study utilizes two nationally representative datasets collected in Ghan and South Africa (SA) during the World Health Organization’s Study on global Ageing and dult health (SAGE-Wave 2) [45]. WHO SAGE is a multinational prospective cohort study that has been conducted in six low and middle-income countries since 2002. The purpose of the study is to examine the health and wellbeing of adults and the ageing process, with the aim of responding to health needs through policy, planning and research. SAGE Wave 2 was conducted in Ghana and SA in 2014/2015 with Wave 3 to be implemented in 2017–2018.

### 2.2. Participants

A total of 10,522 adults were recruited; *n* = 6746 in Ghana and *n* = 3776 in SA. Stratified sampling was conducted to respondents aged 50 years and older, with approximately 30% of adults aged 18–49 years as a comparative cohort. In selecting the sample, all SAGE Wave 1 households were included for SAGE Wave 2 data collection [45]. In SAGE SA, replacements for sample attrition used a systematic sampling approach to randomly select new households as previously described [46]. The sampling method used in SAGE Ghana followed a similar design, based on the 2003 World Health Survey/SAGE Wave 0 [47] with primary sampling units (PSUs) stratified by region and location (urban/rural). Selection of the PSUs was based on proportional allocation by size using the same follow-up and random systematic sampling method as South Africa.

### 2.3. Data Collection

Data collection in Ghana was completed by four field teams each comprising of 3–5 field workers who moved from region to region over an 11-month period (September 2014 to June 2015). In SA, twenty survey teams collected data from respondents across all provinces in the country over a 5-month period (August to December 2015). Surveys were administered in participants’ home language. A computer assisted personal interview was used in collecting the data. All survey teams were trained with support from the WHO SAGE team, with survey teams using standardized training and survey materials [45]. Field teams visited respondents in their homes and workplaces to administer interviews.

### 2.4. Study Measures

The main outcome of the current analysis relates to reported salt KAB captured using a five-item questionnaire adapted from the WHO/PAHO protocol [48]. KAB is the usual term for such research and has been widely used, including in the context of salt behaviours [48,49,50,51,52]. One question investigated knowledge about salt and health—“Do you think that a high salt diet could cause a serious health problem?”—with answer options “yes” or “no”. Another investigated attitudes about salt—“How much salt do you think you consume?”—with answer options of “far too much”, “too much”, “just the right amount”, “too little”, “far too little”, “don’t know”, and “refused”. The congruence between attitudes and perceptions has been explored [53], but for the purposes of comparison with other studies we prefer to retain the terminology of “attitudes”. Three questions assessed salt use behaviours: (1) “Do you add salt to food at the table?”; (2) “In the food you eat at home, salt is added in cooking?”; and (3) “Do you do anything on a regular basis to control your salt or sodium intake?”—with answer options “always”, “often”, “sometimes”, “rarely”, and “never” for items 1 and 2, and “yes”, “no”, “don’t know”, and “refused” for item 3. Likert type response scales were provided, but for analysis of responses to the salt use behaviour questions categories of “always” and “often” were combined to represent “frequent” use, whilst “rarely” and “never” were combined to represent “infrequent” use.

“Currently working” was recorded as “having worked for at least 2 days during the last 7 days”. “Recent use of alcohol” was recorded as “having consumed alcohol in the last 30 days”, while “frequent alcohol intake” was recorded as “having consumed at least one alcoholic drink (on average) one or more days in a week” and “infrequent alcohol intake” was recorded as having consumed at least one alcoholic drink (on average) one to three days per month.

Age, sex, residential location (urban/rural), education, marital status, employment status, alcohol intake, smoking status, blood pressure (BP) and salt behaviour variables were recorded, as shown in Table 1. BP was measured using wrist worn validated Omron BP devices with positional sensors (Omron R6, Kyoto, Japan) [53]. Three BP readings were recorded on the left wrist (1-min between each measurement) while the participant sat with the wrist precisely at the level of the heart and legs uncrossed.

### 2.5. Ethics

Prior to data collection, the study measures were explained to the participants in their home language by the fieldworkers and written informed consent was obtained. The study complied with the ethical principles for medical research involving human subjects as stated in the Declaration of Helsinki [54]. The WHO Research Ethics Review Committee approved the study [RPC149]. Local ethical approval was obtained from the North-West University Human Research Ethics Committee (Potchefstroom, South Africa), the University of the Witwatersrand Human Research Ethics Committee (Johannesburg, South Africa), and the University of Ghana Medical School Ethics and Protocol Review Committee (Accra, Ghana).

### 2.6. Statistical Analysis

All data were collated and analysed using IBM SPSS Statistics for Windows, Version 20.0. (IBM Corp., Armonk, NY, USA). The normality of the data was checked by visual inspection and the Kolmogorov-Smirnov test. Descriptive statistics of frequencies, percentages and median (IQR) were used to describe respondents’ characteristics and responses to survey items. Country differences were evaluated using Chi-square and Independent Samples Mann Whitney *U* tests. The significance level was set at *p* < 0.05. Logistic regression was applied to compare the probability of various salt behaviours between Ghana and SA and odds ratios and 95% confidence intervals (95% CI) were computed. The model was adjusted for potential confounders which included age, sex, residential location, educational level and hypertension prevalence as demonstrated in other studies [52,55,56,57]. BMI was not included in the final regression model as it was not statistically significant.

## 3. Results

Approximately 30% of the recruited samples in both countries had data that could not be retrieved due to technical and data management issues during data collection and retrieval using the CAPI system. This loss of data was non-systematic and the sample size on which the current analysis is based is on a total samples size of *n* = 8145 (by sex). As such, in the sample for Ghana (41.1% (*n* = 1954) were male and 58.9% (*n* = 2799) were female, 75.2% (*n* = 3569) aged 50+ years old and 24.8% (*n* = 1174) aged 18–49 years old) and for SA (32.2% (*n* = 1094) were male and 67.8% (*n* = 2298) were female, 67.5% (*n* = 2291) aged 50+ years old and 32.5% (*n* = 1105) aged 18–49 years old). The characteristics of the population are presented in Table 1. Due to non-responses for some questionnaire items, the number of responses (*n*) for each variable is included in the tables. Significant differences were recorded between the two countries, with more older people (Ghana 75.2%; SA 67.5%; *p* < 0.01) and more men (Ghana 41.1%; SA 32.3%; *p* < 0.01) in Ghana than in SA, whereas SA had more urban residents (Ghana 41.6%; SA 72.8%), and more participants from SA had a higher educational status (Ghana 14.4%; SA 42.1%) and a higher level of hypertension prevalence (SA 44.7%; Ghana 30.9%; *p* < 0.01)

### 3.1. Knowledge

Approximately one-third (31.3%; *n* = 2190) of all respondents were not aware that a high salt diet could cause a serious health problem. This was consistently observed among older and younger adults, men and women and across both countries (Table 2). Significant associations were recorded between respondents’ knowledge and their ethnicity within the South African cohort. Those with a “coloured” ethnic background recorded the highest knowledge (84%, *p* < 0.01).

### 3.2. Attitudes

Three quarters (74.9%; *n* = 5570) of all respondents perceived that they consumed just the right amount of salt. The perception of consuming “just the right amount of salt” was more frequently observed in younger adults, men, and in Ghanaians compared to South Africans

### 3.3. Salt Intake Behaviours

Among all respondents, 18% (*n* = 984) reported that they “always” and “often” (frequently) added salt to food at the table and the majority (*n* = 5999, 91%) reported that they frequently added salt to food at home during cooking. Almost two-thirds (62.4%) reported that they did not take any action to control their salt intake. The response to “taking actions to control salt intake on a regular basis” significantly differed according to knowledge about salt (knowledge and health, *p* < 0.001). For the two countries, fifty-two percent of respondents who did not think high salt could cause health problems reported that they never took actions on regular basis to reduce salt intake. Reported frequent alcohol intake was found to relate to less desirable salt intake behaviours. While 96.7% of respondents reporting frequent alcohol intake (*n* = 1971) also reported always or often adding salt to food while cooking, this behaviour was significantly lower (86.1%) in teetotalers (*n* = 756; *p* < 0.01). Additionally, significantly more teetotalers (51.0%) reported regularly attempting to control their salt intake when compared with frequent drinkers (33.7%; *p* < 0.01). Significant associations were ecorded between respondents who frequently added salt to food at the table and their ethnicity within the South African cohort. Those with African/black ethnic background reported adding more salt to food at the table than any other group (36.5%, *p* < 0.01).

Younger adults more frequently added salt to food at the table, added salt to food eaten at home during cooking and did less on a regular basis to control their salt intake than did older adults (Table 2). More South Africans than Ghanaians reported that they frequently added salt at the table (SA 32.9%; Ghana 9.9%; *p* < 0.001) and did not take actions to control their salt intake (SA 28.8%; Ghana 42.9%). Significantly more Ghanaians than South Africans reported frequently adding salt to food during cooking (SA 79.9%; Ghana 96.3%; *p* < 0.01).

Multivariate analysis adjusted for sex, age, residence, educational level and hypertension prevalence showed that South Africans were more likely than Ghanaians to add salt to food at the table (OR 4.80, CI 4.071–5.611, *p* < 0.001) but less likely to add salt to food at home during cooking (OR 0.16, CI 0.130–0.197, *p* < 0.001) or to take action to control their salt intake regularly (OR 0.44, CI 0.379–0.488, *p* < 0.01); (Table 3). Men and younger adults were also significantly more likely to add salt to food and less likely to control salt intake. Those living in urban areas, educated to high school level or above, or those with hypertension were significantly more likely to regularly control their salt intake.

## 4. Discussion

The finding that 80% or more of adults in Ghana and SA frequently add salt to their food during cooking indicates that discretionary salt use remains high in both countries. Significant differences were observed between the two countries for various behaviours, such as a third of South Africans reported adding salt to food at the table as compared to only a tenth of Ghanaians. Significant differences were also observed between younger and older adults and between the genders. The most potentially detrimental behaviours were identified in men and younger adults. The contribution of discretionary salt intake to total salt intake cannot be under-emphasized even in societies where most salt comes from processed foods. Health promotion activities are needed to decrease individual level salt intake [7] in order to decrease salt preferences over time and thus decrease discretionary salt use [7,36,58].

Knowledge related to the adverse effects of salt on health was poor. Almost one third (*n* = 2190) of both Ghanaians and South Africans were not aware of the relationship between high salt intake and the possibility of a serious health problem. This could potentially explain practices of high discretionary salt use [55,59,60]. Conversely, individuals who know about the health effects of excess salt in the diet have been shown to be more likely to reduce their salt intake [49]. Knowledge of hypertension may influence both salt and self-care behaviours [61]. Our results show that responses to salt knowledge, attitudes and behaviours are significantly related as shown by other authors [56]

Our data indicates participants’ high confidence in their perceived intake of the recommended levels of salt, since three-quarters of respondents reported that they consumed “just the right amount of salt.” A limitation of this study relates to potential under-reporting of salt use, as shown in other studies [49,52,62,63] and responses to salt questions may not reflect actual intakes. Studies that measured 24 h urinary sodium excretion in addition to self-reported discretionary salt use in the same respondents revealed an underestimation in reported salt intake [33,64]. Hypertension levels were high in both samples but we did not investigate whether hypertensive individuals that were receiving treatment to control their blood pressure had also received advice on salt intake. There is a possibility of misreporting, as those who have received information on salt use may feel that they consume the recommended amount. Furthermore, there might be some lack of knowledge relative to the amount of salt recommended for daily consumption, as was the case in other studies [56]. This discrepancy between perceived and actual salt consumption has the potential to result in higher than anticipated salt intakes [65].

Our data indicates a need to intensify consumer education on salt intake awareness in both countries, including discretionary salt use. Previous research contributing to South African policy change [33] suggests that further investigation into particular food items that contribute high amounts of salt to the diet is needed in Ghana. Consistent with other studies [56], more attention should be directed towards the younger population (18–49 years old) and to men, who reported worse discretionary salt knowledge, attitudes and behaviours. Dietary salt education and awareness should also be integrated into school curricular and youth programmes, particularly as hypertension and associated morbidity and mortality typically occur at younger ages in Sub Saharan Africa (SSA) [66,67].

Our finding that Ghanaians add more salt to food at home during cooking compared with their South African counterparts may not be surprising given investment in campaigns to increase consumption of iodized salt [68,69,70]. Within Ghana, any action related to salt reduction for addressing NCDs appears to have been overshadowed by the strong focus on the prevention of iodine deficiencies [39,40,41,70]. This situation appears in contrast to WHO guidelines [14], which suggest compatibility of polices on salt reduction and salt iodisation. The guidelines advise that regular monitoring of sodium (salt) intake and iodine intake at country level is needed to adjust salt iodisation over time to ensure that individuals consume sufficient iodine while reducing overall intake of salt. If salt is sufficiently iodised, a reduction of salt intake to the recommended level of 5 g per day should still provide an adequate amount of iodine [43]. Ghana’s strong emphasis on the reduction of iodine deficiencies provides a unique and untapped opportunity for addressing discretionary salt use and behaviour change. Salt used in food processing is not included in the mandatory Universal Salt Iodisation policies in either Ghana or South Africa, although there is some evidence from South Africa that indicates up to a third of margarines, bread, and savoury snack seasonings contain iodised salt [71].

We found that South Africans were almost 5 times more likely than Ghanaians to add salt to food at the table (controlling for sex, age, location, education level and hypertension prevalence). This finding was expected, as a greater proportion of the cohort in SA lived in urban areas compared with Ghana. Urbanization is one of the key drivers for excess salt consumption [15]. Further investigation regarding the effect of acculturation on addition of salt to food is of interest. The survey was conducted prior to salt legislation being introduced in SA, therefore it will be of importance to determine how the lowering of salt in processed foods impacts on both discretionary salt behaviours and actual salt intake levels in SA in the next wave of SAGE in 2017–2018.

The present study shows that almost two-thirds of the population were not taking any action to control their salt intake, and this may be explained by the finding that a third of respondents did not have any knowledge regarding the links between high salt intake and health problems. Among the key broad strategies for salt reduction is individual responsibility and action [7], for example, limiting the consumption of salty snacks and reducing the amount of salt used in cooking. The Health Belief Model explains that perceived seriousness, perceived susceptibility, perceived benefits and perceived barriers are critical evaluations an individual undertakes prior to engaging in a health behaviour [72]. Our data suggests that strategies are required to firstly increase awareness within the Health Belief Model of health promotion as a means of supporting behaviour change. The other broad strategy is at a systemic level—including measures like those adopted in South Africa to work with food manufacturers to lower salt levels in processed foods.

The findings of the present study were somewhat surprising because, despite the extensive public awareness campaign in SA to address population salt intake levels and influence salt related behaviour (Salt Watch) [37,73] and despite that population reporting greater salt knowledge, it was the Ghanaians who appeared to be more actively controlling their salt intake (Table 2). This highlights the point that knowledge alone is not sufficient to cause a change in behaviour. Consistent with the present study, research from Newson et al. (2013) [62] showed that almost half of survey respondents were not interested in reducing their salt intake and had no intention of making any changes to their salt consumption in the immediate future. With a large hypertension burden [24], it is likely that some South Africans may not feel empowered to make dietary changes. Additionally, cultural beliefs and practices that encourage salt consumption are also of concern. Salt use for spiritual and religious purposes is common place in SA [37] and this would need to be considered in health messages. 

The finding that frequent alcohol intake was associated with less desirable salt intake behaviours has been reported by others [74]. Differences in reported alcohol intake between Ghanaian and South African study participants are similar to previous analyses of alcohol consumption from Wave 1 of SAGE which indicated that there were fewer “never” drinkers (lifetime abstainers) in Ghana than in SA, while the South African cohort had more “at risk” drinkers [75].

It is well established that reducing dietary salt to 3 g/day leads to a reduction in blood pressure in both in hypertensives and normotensives [76]. Given the current estimated prevalence of hypertension in the African region, it is imperative that salt reduction messages are included in public health campaigns alongside various strategies to support behaviour change while also modifying salt levels in commonly consumed foods. Provision of practical skills and strategies to encourage change in salt intake behaviours is key in this regard.

The present study had several strengths which included the use of large, nationally representative sample sizes in both SA and Ghana. A potential limitation relates to the oversampling of older people in keeping with the purpose of the SAGE, which may limit generalizability of the findings to the population younger than 50 in both countries. Regardless, these are relatively large representations of the 50+ population, with younger comparison groups in both countries.

## 5. Conclusions

This study provided important insights into salt knowledge, attitudes and behaviours (KAB) in SSA. High discretionary salt use remains common practice in the region and needs urgent attention in the face of high and rising hypertension levels. Significant differences in salt KAB were evident between the two countries which suggests the use of different strategies and approaches for combatting high discretionary salt intake may be appropriate, although strategies particularly targeting men and younger adults may be beneficial for both countries. The findings suggest that SA requires investment into public health campaigns to address the practice of adding salt to foods at the table, while in Ghana a focus on changing behaviours related to the use of salt in cooking is required. Campaigns and consumer education strategies—based on the Health Belief Model—may be useful to raise awareness for salt reduction in SSA.

## Figures and Tables

**Table 1:** Sociodemographic characteristics and selected health characteristics of the study samples in Ghana and South Africa, Study on Global Ageing and Adult Health (SAGE) Wave 2.

Characteristics	Age Categories—Countries Combined			Ghana *n* = 4753	South Africa *n* = 3392	*p* Value
	18–49 Years Old (*n* = 2279)	50+ Years Old (*n* = 5860)	*p* Value			
Age in years, median (IQR) 50 plus years, *n* (%)				*n* = 4743 58 (19.0) 3569 (75.2)	*n* = 3396 54 (24.0) 2291 (67.5)	<0.01 <0.01
Sex male, *n* (%)	*n* = 2277 871 (38.3)	*n* = 5857 2171 (37.0)	0.32	*n* = 4753 1954 (41.1)	*n* = 3392 1094 (32.3)	<0.01
Residence urban, *n* (%)	*n* = 2110 1176 (55.7)	*n* = 5560 2918 (52.5)	0.01	*n* = 4728 1970 (41.6)	*n* = 2924 2124 (72.8)	<0.01
Education						
Ever attended school, *n* (%)	*n* = 2108 1839 (87.2)	*n* = 5546 3327 (60.0)	<0.01	*n* = 4734 2764 (58.4)	*n* = 2920 2402 (82.3)	<0.01
Educational level high school or above, *n* (%)	*n* = 2099 1164 (55.5)	*n* = 5493 1646 (30)	<0.01	*n* = 4706 678 (14.4)	*n* = 2886 696 (24.1)	<0.01
Employment status: currently working, (%)	*n* = 1473 1124 (76.3)	*n* = 4832 2611 (54.0)	<0.01	*n* = 4537 3169 (69.8)	*n* = 1768 566 (32.0)	<0.01
Marital status: married/cohabiting, *n* (%)	*n* = 937 231 (24.7)	*n* = 1984 684 (34.5)	<0.01	*n* = 4738 2692 (56.8)	*n* = 2921 915 (31.3)	<0.01
Waist to height ratio <0.5, *n* (%)	*n* = 1853 693 (37.4)	*n* = 4807 1369 (28.5)	<0.01	*n* = 4347 1553 (35.7)	*n* = 2313 509 (22.0)	<0.01
Body Mass Index (BMI), median (IQR)	*n* = 1904 25 (7.7)	*n* = 4883 24.2 (8.4)	<0.01	*n* = 4456 22.9 (6.2)	*n* = 2331 28.6 (9.9)	<0.01
Alcohol intake						
Never *n* (%)	*n* = 1597 397 (24.0)	*n* = 1625 400 (24.6)	0.87	*n* = 1168 786 (67.3)	*n* = 2054 1639 (80.0)	<0.01
Recently yes, *n* (%)	*n* = 515 351 (68.2)	*n* = 1379 912 (66.1)	0.41	*n* = 1379 916 (66.4)	*n* = 515 347 (67.4)	0.69
Frequent, *n* (%)	*n* = 508 203 (40.0)	*n* = 1371 595 (43.4)	<0.01	*n* = 1369 635 (46.4 )	*n* = 510 163 (32.0 )	<0.01
Smoking status, current use of tobacco, *n* (%)	*n* = 1616 137 (8.5)	*n* = 1884 474 (25.2)	<0.01	*n* = 1340 208 (15.5)	*n* = 2160 403 (18.7)	0.06
Systolic blood pressure mmHg, median (IQR)	*n* = 2030 119 (20.0)	*n* = 5370 131 (28.0)	<0.01	*n* = 4674 124.67 (28.0)	*n* = 2726 130.7 (26.0)	<0.01
Diastolic blood pressure mmHg, median (IQR)	*n* = 2030 75.7 (15.0)	*n* = 5370 79.3 (17.0)	<0.01	*n* = 4674 77 (16.0)	*n* = 2726 80.67 (16.0)	<0.01
Hypertensive, *n* (%)	*n* = 2079 401 (19.3)	*n* = 5455 2320 (42.5)	<0.01	*n* = 4675 1444 (30.9)	*n* = 2859 1277 (44.7)	<0.01

Data recorded as median (inter quartile range) and frequencies (%). Smokers identified by self-report. Frequent alcohol use defined as the consumption of one or more alcoholic drinks a day/week. Hypertensive categorized as BP ≥ 140/90 mmHg or previous diagnosis. Continuous variables were compared using the Independent Samples Mann Whitney U test; categorical variables were compared using the Chi-Square test.

**Table 2:** Association between salt knowledge, attitudes and behaviours (KAB) and demographic characteristics of Ghanaians (*n* = 6746) and South Africans (*n* = 3776); younger (*n* = 2279) and older (*n* = 5860); men (*n* = 3048) and women (*n* = 5860), SAGE Wave 2.

	Age Category—Countries Combined			Sex—Countries Combined			Ghana	SA	*p* Value
	18–49 Years Old *n* = 2279	50+ Years Old *n* = 5860	*p* Value	Men *n* = 3048	Women *n* = 5097	*p* Value	*n* = 4753	*n* = 3392	
Do you think that a high salt diet could cause a serious health problem? Yes *n* (%)	*n* = 1940 1309 (67.5)	*n* = 5061 3502 (69.2)	0.16	*n* = 2602 1756 (67.5)	*n* = 4399 3055 (69.4)	0.09	*n* = 4328 2920 (67.5)	*n* = 2673 1891 (70.7)	<0.01
How much salt do you think you consume? Just the right amount, *n* (%)	*n* = 2047 1613 (78.8)	*n* = 5392 3957 (73.4)	<0.01	*n* = 2804 2176 (77.6)	*n* = 4635 3394 (73.2)	<0.01	*n* = 4622 3510 (75.9)	*n* = 2817 2060 (73.1)	<0.01
Do you add salt to food at the table? “Always” and “Often” *n* (%)	*n* = 1453 311 (21.4)	*n* = 4026 673 (16.7)	<0.01	*n* = 2024 393 (19.4)	*n* = 3455 591 (17.1)	0.31	*n* = 3552 350 (9.9)	*n* = 1927 634 (32.9)	<0.01
In the food you eat at home, salt is added in cooking…? “Always” and “Often” *n* (%)	*n* = 1822 1689 (92.7)	*n* = 4771 4310 (90.3)	0.03	*n* = 2552 2374 (93)	*n* = 4041 3625 (89.7)	<0.01	*n* = 4459 4294 (96.3)	*n* = 2134 1705 (79.9)	<0.01
Do you do anything on a regular basis to control your salt or sodium intake? Yes *n* (%)	*n* = 1990 688 (34.6)	*n* = 5249 2034 (38.8)	0.01	*n* = 2718 970 (35.7)	*n* = 4521 1752 (38.8)	<0.01	*n* = 4518 1939 (42.9)	*n* = 2721 783 (28.8)	<0.01

Note: “All” represents respondents from both countries. Data was recorded in frequencies. Chi square tests were conducted.

**Table 3:** Associations between sociodemographic variables and salt behaviours of adults in Ghana (*n* = 6746) and South Africa (*n* = 3776)—comparing the odds ratio for sub-optimal salt behaviours, SAGE Wave 2.

Variables	Salt Frequently Added to Food at the Table	Salt Frequently Added to Food during Cooking	Takes Regular Action to Control of Salt Intake
	OR (95% CI)	OR (95% CI)	OR (95% CI)
Ghana	Referent	Referent	Referent
SA	4.80 (4.071–5.611) *	0.16 (0.130–0.197) *	0.436 (0.379–0.488) *
Female	Referent	Referent	Referent
Male	1.40 (1.182–1.605) *	1.36 (1.118–1.654) *	0.78 (0.718–0.884) *
18–49 years old	Referent	Referent	Referent
50+ years old	0.78 (0.655–0.920)*	0.56 (0.450–0.711) *	1.23 (1.096–1.384) *
Rural	Referent	Referent	Referent
Urban	0.89 (0.762–1.041)	0.90 (0.744–1.099)	1.41 (1.271–1.565) *
Primary school and below	Referent	Referent	Referent
High school and above	0.87 (0.741–1.020)	0.86 (0.710–1.048)	1.12 (1.004–1.240) *
Normotensive	Referent	Referent	Referent
Hypertensives	1.11 (0.952–1.299)	0.88 (0.734–1.067)	1.48 (1.328–1.649) *

Note: Referent indicates reference category used for the comparison. Hypertension was categorized as BP ≥ 140/90 mmHg or previous diagnosis, logistic regression was adjusted for sex, age category, residence, educational level and hypertension prevalence, * *p* < 0.05.
